# Application of 3D printing technology in the surgical management of advanced ovarian serous carcinoma: a case report

**DOI:** 10.3389/fonc.2025.1541919

**Published:** 2025-02-28

**Authors:** Hang Xi, Chunhui Liu, Nana Tian, Min Geng, Jinfang Hu, Haohao Li, Wenbo Pan, Xiaohong Sun, Zhihui Cai, Shasha Bai

**Affiliations:** ^1^ Gynecology department, the Affiliated Hospital of Hebei University, Baoding, Hebei, China; ^2^ Gynecology department, Yi County Hospital, Baoding, Hebei, China

**Keywords:** 3D printing, ovarian serous carcinoma, gynecological surgery, surgical planning, precision medicine, anatomical modeling, patient-specific surgery

## Abstract

Advanced ovarian serous carcinoma presents significant surgical challenges due to tumor size, deep location, and complex anatomical relationships with surrounding structures. This case report highlights the use of 3D printing technology to improve surgical planning and outcomes in such complex scenarios. A 48-year-old female presented with bilateral ovarian tumors, confirmed as serous cystadenocarcinoma. Preoperative 3D modeling was employed to create a detailed anatomical model based on imaging data. This model provided precise visualization of tumor size, vascular supply, and relationships with adjacent organs, facilitating the development of an optimal surgical plan. During surgery, bilateral ovarian tumors were resected along with the uterus, omen tum, and lymph nodes, achieving complete R0 resection. Postoperatively, the patient recovered well, with no complications or recurrence observed during follow-up. This case underscores the value of 3D printing in enhancing surgical precision and safety in complex gynecological oncology cases. By providing individualized anatomical insights, 3D printing supports preoperative planning, improves patient outcomes, and contributes to advancing precision medicine in surgical practice.

## 1 Introduction

Ovarian serous carcinoma is the most common and aggressive type of ovarian epithelial malignancy, characterized by complex biological behavior and a propensity for early dissemination to the pelvic and abdominal cavities (1). Due to its anatomical location deep within the pelvis, combined with the involvement of adjacent organs (e.g., uterus, fallopian tubes, bladder, and rectum) and major blood vessels, surgical resection poses significant challenges (2). Although conventional preoperative imaging modalities such as ultrasound, CT, and MRI provide some anatomical information, they are limited in accurately assessing the three-dimensional spatial relationships between the tumor and surrounding tissues (3, 4). These limitations may increase intraoperative risks and complications.

In recent years, three-dimensional (3D) printing technology has emerged as a promising adjunct in the field of surgery, attracting increasing attention for its potential to enhance clinical outcomes (5). By converting patient-specific imaging data into high-resolution, tangible anatomical models, 3D printing facilitates precise visualization of the spatial relationships between tumors and adjacent structures. This technology plays a pivotal role in preoperative planning and intraoperative guidance, particularly in gynecologic oncology (6). Its application enables surgeons to achieve a more comprehensive understanding of complex pelvic anatomy, optimize surgical strategies, and improve the completeness of tumor resection while minimizing collateral damage to surrounding healthy tissues (7, 8).

However, the specific application of 3D printing technology in ovarian serous carcinoma surgeries, particularly in complex cases, remains inadequately documented. This case report presents a patient with advanced ovarian serous carcinoma characterized by extensive tumor invasion of multiple pelvic and abdominal structures, posing significant surgical challenges. An individualized 3D-printed pelvic anatomical model was constructed to precisely evaluate the spatial relationships between the tumor and critical surrounding organs and vasculature, successfully facilitating surgical planning and execution. Through an in-depth analysis of this case, we aim to explore the clinical utility of 3D printing technology in complex ovarian serous carcinoma surgeries, highlighting its advantages in enhancing surgical safety and efficacy. This report seeks to provide a reference for managing similar cases in the future.

## 2 Case presentation

### 2.1 Clinical examination

The patient, Ms. Liu, a 48-year-old female, was admitted due to persistent lower abdominal distension accompanied by mild abdominal pain lasting for one month. Approximately one month prior, the patient experienced a progressive sense of abdominal fullness without apparent precipitating factors, accompanied by reduced appetite but no nausea, vomiting, or other gastrointestinal symptoms. Over the past week, her symptoms worsened, with intermittent mild abdominal pain. No fever, weight loss, or other significant systemic symptoms were reported. The patient had a history of regular menstrual cycles, with her last menstruation approximately three months ago. She is unmarried and nulliparous.

Upon admission, a gynecological examination revealed a mixed cystic-solid mass on the right side of the pelvis, approximately 11 cm in diameter. A similar cystic-solid mass, measuring about 10 cm in diameter, was palpated in the left adnexal region. Both masses had poorly defined borders, limited mobility, and were fixed in position. The high placement of the masses obscured the uterus, making palpation suboptimal. Bimanual examination showed no significant abnormalities, with no thickening of the uterosacral ligaments bilaterally. The rectal mucosa was smooth, and the glove withdrawn during rectal examination showed no blood staining. No other notable abnormalities were detected. The patient denies any prior surgical history, as well as a history of hypertension or diabetes.

To further clarify the diagnosis, the patient underwent multiple imaging studies. Transvaginal ultrasound (performed on August 16, 2023) revealed an anteverted uterus measuring approximately 5.5 × 4.9 × 4.5 cm, with heterogeneous myometrial echogenicity. Multiple hypoechoic nodules were observed within the myometrium, the largest located in the posterior wall measuring approximately 2.4 × 1.9 × 1.6 cm. The endometrial thickness was approximately 0.5 cm, and an intrauterine device was noted with normal positioning. In the left adnexal region, a mixed cystic-solid mass was identified, measuring approximately 9.4 × 8.4 × 8.2 cm. The mass was predominantly solid, with relatively well-defined borders, an irregular lobulated shape, and demonstrated peripheral and internal blood flow signals on color Doppler flow imaging (CDFI), with a resistance index (RI) of 0.57. Similarly, a mixed cystic-solid mass was detected in the right adnexal region, measuring approximately 11.3 × 10.2 × 7.6 cm. This mass also displayed relatively well-defined borders, an irregular lobulated contour, and significant blood flow signals both peripherally and internally on CDFI, with an RI of 0.45. Additionally, a free hypoechoic area consistent with fluid accumulation was observed in the pelvic cavity, with a depth of approximately 5.4 cm, indicating the presence of ascites in the pelvic and abdominal cavities.

Regarding tumor markers, preoperative evaluation showed an elevated CA125 level of 69.60 U/ml (reference value: 0-35 U/ml), while the remaining tumor markers, including CEA, CA15.3, CA19.9, and HE4, were within normal limits.

**Figure 1:**
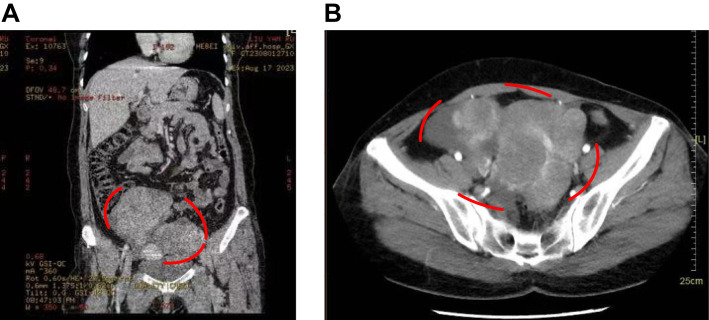
Patient’s CT images. **(A)** CT showed a cystic-solid masses in the adnexal area. **(B)** Enhanced CT indicated the marked location was a cystic-solid masses in the adnexal area, with a high possibility of malignancy.

Based on imaging findings, the patient was initially diagnosed with bilateral adnexal cystic-solid masses, with a high suspicion of malignancy, particularly ovarian cancer, given the masses’ irregular morphology, vascular characteristics, and the presence of pelvic ascites. Subsequent contrast-enhanced pelvic and abdominal CT further supported this diagnosis, revealing bilateral adnexal masses consistent with malignancy, significant pelvic and abdominal ascites, and multiple hepatic cysts (**Figure 1**). Additionally, a small nodule in the right lung was noted, for which follow-up was recommended. The intrauterine device was confirmed to be in a normal position. Colonoscopy showed no abnormalities in the colon or rectum, while gastroscopy indicated chronic non-atrophic gastritis without malignancy.

The patient was fully informed preoperatively and, after thorough discussion with her family, consented to surgical treatment. Based on the comprehensive evaluation of imaging findings, clinical symptoms, and physical examination, along with a strong suspicion of malignancy, the final diagnosis was bilateral ovarian serous cystadenocarcinoma. Given the large size of the ovarian tumors and their dense adhesion to surrounding organs, the surgery was deemed highly challenging. To enhance precision and safety, 3D printing technology was employed preoperatively to assist in surgical planning.

### 2.2 3D modeling and preoperative planning

**Figure 2:**
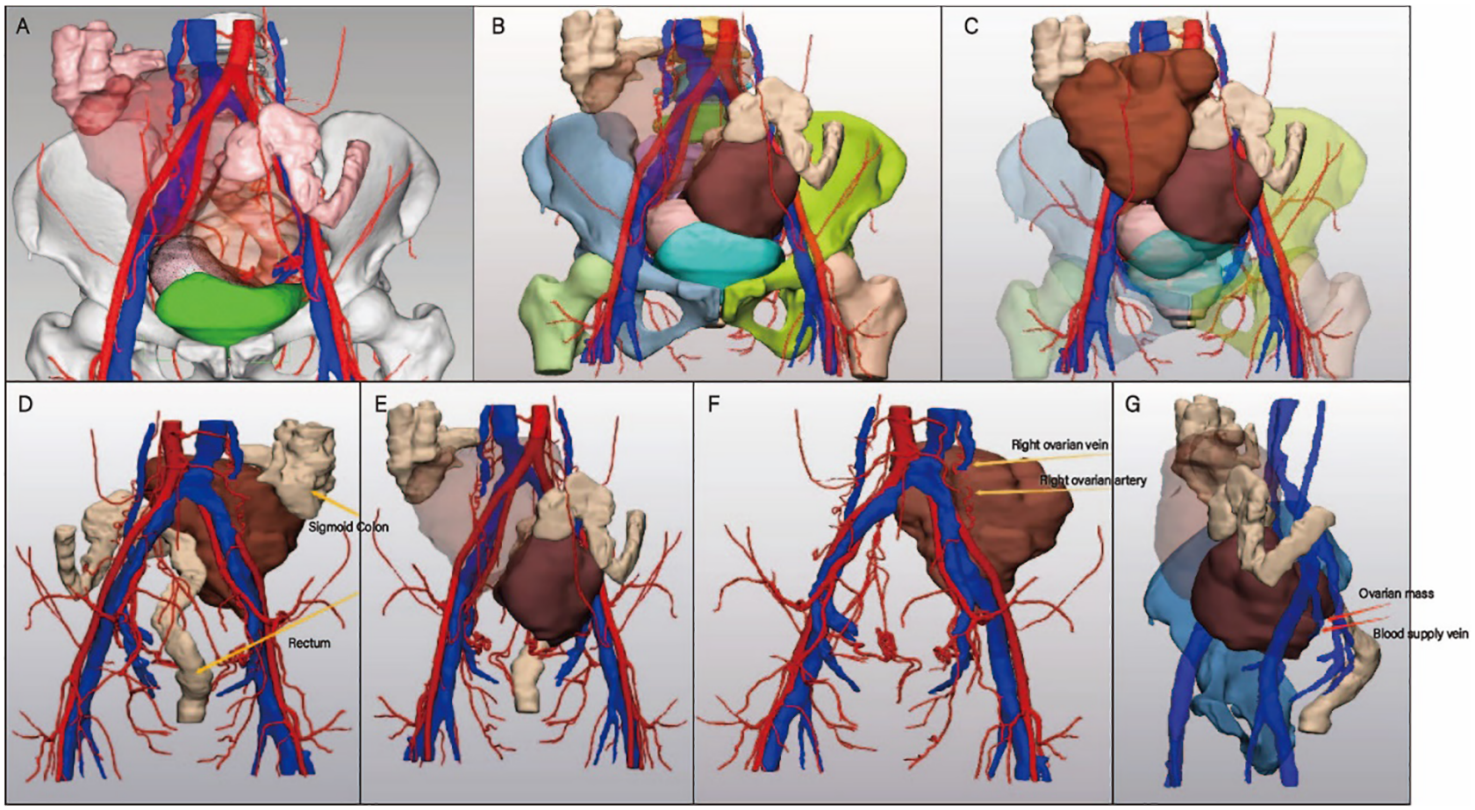
3D reconstruction and 3D printed model of patient. **(A-C)** Key views of the 3D modeling. **(A)** Demonstrates the spatial relationship between the abdominal mass and pelvic organs. **(B)** Displays the main vascular pathways with part of the mass hidden for better visualization. **(C)** Illustrates vascular encapsulation and trajectories with the mass fully visualized. **(D-G)** Highlights critical points in surgical planning. **(D)** Shows the spatial relationship between the mass, sigmoid colon (upper arrow), and rectum (lower arrow). **(E)** Further reveals the positional relationship of the mass with the sigmoid colon and rectum. **(F)** Highlights the right ovarian vein (upper arrow) and right ovarian artery (lower arrow). **(G)** Marks the ovarian mass (upper arrow) and its blood supply vein (lower arrow).

Preoperatively, collaboration with the hospital’s 3D printing center enabled the utilization of imaging data for three-dimensional reconstruction, providing a precise depiction of the spatial relationships between the tumors and surrounding anatomical structures. The enhanced CT scan was performed using the Revolution CT (GE Healthcare, Milwaukee, USA), with a single breath-hold scan covering the area from the diaphragm to the pubic symphysis. The obtained CT images were imported in DICOM format into MIMICS 23.0 software (Materialise NV, Leuven, Belgium), where window adjustments were made to optimize image reconstruction. The generated STL files were further processed in 3-matics 15.0 software (Materialise NV, Leuven, Belgium) before being imported into the E3D digital medical modeling and design software (Central South University, China) and uploaded to a cloud platform. The processed model was then printed using an SLS 3D printer (China Yingpu). Flexible TPU material was used for printing the arteries, veins, and tumors, while high-strength, wear-resistant nylon material was used for the bones. A combination of rigid and flexible printing techniques was employed to create the final ovarian tumor model, ensuring both structural integrity and anatomical accuracy. The 3D modeling clearly illustrated the size, location, and vascular characteristics of the bilateral ovarian tumors, offering critical guidance for surgical planning (**Figure 2**).

The reconstructed images revealed distinct vascular and anatomical characteristics of the bilateral ovarian tumors. The blood supply to the right ovarian tumor originated from a higher position, located on the posterior aspect of the tumor, with prominent, large-caliber vessels and rich blood flow. In contrast, the left ovarian tumor’s blood supply was positioned lower, with feeding vessels located on the lower posterior aspect of the tumor and a more complex vascular course. The sigmoid colon was compressed and deformed by the bilateral tumors, occupying the space between them, with partial displacement of the colonic origin. The upward pressure exerted by the right tumor caused morphological changes in a segment of the colon. Additionally, fluid accumulation was observed in the rectouterine pouch, and the uterus was obscured by the tumors, making direct palpation impossible.

Based on the guidance provided by 3D modeling, the following surgical steps were planned preoperatively. Right Ovarian Tumor: Given the higher origin of its blood supply, resection of the feeding vessels for the right ovarian tumor was anticipated to be relatively straightforward. The plan was to first ligate and transect the feeding vessels of the right tumor, followed by the resection of the right ovarian tumor. Pelvic Space Management: The removal of the right ovarian tumor was expected to release sufficient pelvic space, thereby facilitating better exposure of the left ovarian tumor. This approach aimed to provide adequate operative space, reducing the technical challenges and risks during the subsequent steps. Left Ovarian Tumor: Due to its deeper location and displacement by the adjacent intestinal and other structures, precise resection of the left ovarian tumor was planned with meticulous attention to avoiding damage to surrounding tissues. The surgical strategy emphasized minimizing intraoperative risks while ensuring complete tumor removal.

### 2.3 Surgical procedure

Under general anesthesia, the patient was positioned and underwent exploratory laparotomy. Intraoperative findings revealed bilateral ovarian tumors, each approximately 10 cm in diameter, with dense adhesions to surrounding structures, particularly the sigmoid colon and bowel. The pelvic cavity was almost entirely filled by the tumors, making the uterus impalpable. Following preoperative 3D modeling guidance, the surgery proceeded as follows:

1. **Right Ovarian Tumor Resection:** The lateral peritoneum of the right ovary was incised, and the tumor was gradually dissected from the surrounding tissues. The right ureter was carefully identified and preserved. The ovarian arteries and veins, noted to be large and with significant blood flow, were isolated. After high ligation of the feeding vessels, the right ovary and tumor were excised, which significantly released pelvic space.
2. **Left Ovarian Tumor Resection:** Using a similar approach, the lateral peritoneum of the left ovary was incised. The left ureter was identified and preserved, and the tumor was meticulously dissected, ensuring the integrity of adjacent critical structures. The left ovary and its feeding vessels were subsequently excised.
3. **Further Resections:** Intraoperative frozen pathology confirmed malignancy in both ovarian tumors, consistent with serous carcinoma. As a result, additional surgical procedures were performed, including hysterectomy, omentectomy, and pelvic and para-aortic lymphadenectomy.

The surgery lasted approximately 3 hours and 37 minutes, with an estimated intraoperative blood loss of 350 mL. R0 resection was achieved, with complete tumor removal and no evidence of significant damage to major blood vessels.

### 2.4 Postoperative outcomes

The postoperative pathological report confirmed Stage IIA bilateral ovarian serous cystadenocarcinoma. The patient experienced an uneventful recovery and was discharged on postoperative day 7 in good condition. No intraoperative or postoperative complications were observed. Following discharge, the patient underwent adjuvant chemotherapy consisting of paclitaxel and carboplatin, with one cycle administered every 28 days for a total of 6 cycles. The patient demonstrated favorable recovery during follow-up, with no signs of recurrence or metastasis. Postoperatively, lifelong follow-up is recommended, with follow-up exams every 3 months for the first 3 years, every 6 months from years 3 to 5, and annually after 5 years.

## 3 Discussion

3D printing technology, as an innovative auxiliary tool, has gained widespread application in the medical field in recent years. It has shown tremendous potential, particularly in surgical planning, medical education, patient counseling, and the development of personalized prosthetics and implants (9, 10). This case report highlights the practical application of 3D printing technology in guiding the surgical treatment of ovarian serous carcinoma. It further explores its advantages in clinical practice and discusses potential directions for its future development.

### 3.1 Surgical planning and training

One of the primary applications of 3D printing technology is the creation of patient-specific anatomical models for preoperative planning. These models provide intuitive and detailed visual information, enabling surgeons to better understand complex anatomical structures and improve surgical precision and safety. For instance, in microvascular decompression (MVD) surgeries, 3D-printed models assist surgical teams in gaining a deeper understanding of patient-specific anatomy during preoperative planning, thereby reducing intraoperative risks (11). In a study where digital anatomical models were created using 3D printing technology and tested across surgeons with varying experience levels, it was demonstrated that the use of these models enhanced surgical planning and significantly reduced surgical risks for patients (12). These findings underscore the value of 3D printing in enhancing surgical outcomes and optimizing preoperative strategies.

In the study by Youn JK et al., the effectiveness of understanding retroperitoneal tumors through three methods—2D CT images, 3D reconstructions, and 3D-printed models—was compared. The results demonstrated that 3D-printed models offered the highest level of comprehension (13). This suggests that the use of 3D printing can enhance understanding of pathological conditions, particularly in pediatric patients, enabling healthcare providers and guardians to better grasp the nature of the disease, thereby improving the informed consent process. Additionally, 3D models have proven valuable in guiding treatment decisions for cervical cancer patients by offering a clearer understanding of their disease. Recent advancements have also seen the development of predictive models for lymph node metastasis and survival outcomes following radical hysterectomy in cervical cancer. These models frequently incorporate 3D modeling techniques to better represent tumor characteristics, such as volume and contour (14–16). Furthermore, as attempts to expand the indications for fertility-sparing surgery in cervical cancer continue, 3D models are becoming increasingly useful as navigational tools and as resources for further research (17).

In medical education, 3D-printed models play a pivotal role in enhancing learning outcomes. In dentistry, studies have shown that 3D printing workflows can be employed to design and produce customized training models for dental schools, providing highly realistic simulation environments (18). Similarly, 3D-printed skeletal structures have been successfully used in anatomy education, helping students gain a deeper understanding of complex anatomical features (19).One study demonstrated that 3D-printed models facilitate spatial visualization, potentially improving students’ comprehension and satisfaction in diagnosing and treating rib dysfunctions, making them an effective teaching aid (20). Beyond dentistry and orthopedics, 3D-printed anatomical models have been extensively used in cardiothoracic surgery, otolaryngology, urology, and other specialties. These models allow students and trainees to practice surgical techniques in a risk-free environment, thereby enhancing their procedural skills. This practical approach has significantly improved the quality of medical education, providing future physicians with a more tangible and immersive learning experience (21–24).

In this case, the patient’s bilateral ovarian tumors were large, deeply located, and had complex relationships with surrounding tissues, significantly increasing the difficulty and risk of surgery. By employing 3D printing technology, the surgical team obtained a three-dimensional anatomical model of the patient’s pelvis, providing detailed insights into the size, location, vascular supply, and spatial relationships of the tumors with adjacent organs. This enabled more precise preoperative planning, facilitated the development of an optimal surgical strategy, reduced intraoperative uncertainties, and enhanced both the success rate and safety of the procedure.

### 3.2 Patient education

Extensive research has demonstrated that improving communication between patients and healthcare providers enhances treatment adherence, facilitates more effective recovery, and promotes better emotional well-being post-discharge (25–27). Patient-centered care has long been a cornerstone of clinical practice, and the advent of 3D printing technology has further empowered patients to participate actively in decision-making and take greater control of their health. By providing intuitive 3D models, patients can gain a clearer understanding of their condition and the planned treatment, thereby alleviating anxiety and boosting confidence in their care plan.

In this case, the 3D-printed model was utilized not only for surgical planning but also as a tool for communication with the patient and their family. By visually demonstrating the model, the patient gained a deeper understanding of their condition, the necessity of the surgery, and its potential risks. This sense of involvement enhanced the patient’s treatment adherence and contributed to postoperative recovery. Studies have shown that when patients fully comprehend their condition and treatment plan, they are more likely to establish trust with healthcare providers and actively cooperate with treatment (28–30).

### 3.3 Development of personalized prosthetics and implants

3D printing has demonstrated exceptional capabilities in the creation of personalized prosthetics and implants, enabling customization based on the patient’s specific anatomical structure. This tailored approach ensures a better fit, reduces postoperative complications, and enhances patient outcomes (31). For instance, 3D-printed knee prosthetics designed using CT scan data have been shown to improve patient comfort and mobility. In radiation therapy for cervical cancer, 3D printing also plays a critical role. Radiation therapy is the standard treatment for locally advanced cervical and vaginal cancers, as well as for certain primary and recurrent gynecologic malignancies. However, conventional applicators often fail to effectively cover the target volume due to anatomical constraints or variations in tumor size and shape. 3D printing technology enables the production of customized brachytherapy applicators that conform precisely to the patient’s anatomy, improving treatment precision and efficacy while minimizing damage to surrounding healthy tissues (32).

### 3.4 Limitations and future prospects

Despite its immense potential, 3D printing technology in medicine still faces several limitations. Firstly, the prolonged printing time and high costs may hinder its widespread clinical application (32). Secondly, the precision of 3D printing and the performance of available materials require further improvement to meet the demands of complex medical applications. Moreover, the strict requirements for biocompatibility and safety in the medical field necessitate more rigorous selection and regulation of 3D printing materials.

With advancements in technology and the development of novel materials, the future of 3D printing in medicine is promising. It holds potential for greater contributions to precision medicine, the formulation of individualized treatment plans, and patient education, offering more efficient and personalized solutions for clinical care. In the field of obstetrics and gynecology, 3D printing not only supports complex surgical procedures but may also provide new research directions and therapeutic approaches in areas such as reproductive medicine and endocrine disorders.

## 4 Conclusion

This case highlights the considerable value of 3D printing technology in complex gynecological tumor surgeries. By employing personalized anatomical models, the surgical team was able to plan the procedure with high precision, minimizing intraoperative risks and improving both surgical outcomes and patient satisfaction. At our hospital, 3D reconstruction and printing technology has been in clinical use for five years, with broad applications across departments including orthopedics, thoracic surgery, gynecology, urology, and hepatobiliary surgery, while ongoing exploration into its use in neurosurgery and dentistry is underway. The technology requires minimal equipment investment, with costs primarily associated with software licensing and graphic workstations. Currently, the cost of 3D reconstruction is approximately $400, and we are actively optimizing alternative materials and processes to reduce costs. Within the next year, we expect to reduce the cost of 3D printing to $280 and improve processing time to 24 hours. The widespread application of this technology across multiple oncology centers is within reach, paving the way for more individualized and precise healthcare.

## Data Availability

The original contributions presented in the study are included in the article/Supplementary Material. Further inquiries can be directed to the corresponding author/s.
